# Dissociations between data-driven and goal-driven effort reports: Performance, metacognition, and affect

**DOI:** 10.1177/17470218231186609

**Published:** 2023-07-31

**Authors:** Kate Van Kessel, Michelle Ashburner, Evan F Risko

**Affiliations:** Department of Psychology, Faculty of Arts, University of Waterloo, Waterloo, Ontario, Canada

**Keywords:** Effort, metacognition, learning, affect

## Abstract

Measuring effort has long been a challenge and this seems particularly true in the case of subjective effort. Koriat et al. compared two types of effort frames, what they call data-driven effort, the amount of effort perceived to be required by a task, and goal-driven effort, the amount of effort one chooses to invest in a task. This study investigates whether self-reports of data- and goal-driven effort are differentially associated with test performance, metacognition, and affect in a complex learning task. Results demonstrate that data- and goal-driven effort have qualitatively different relations with many of these variables. For example, partial correlations revealed data-driven effort was negatively associated with prospective and retrospective performance estimates, but the opposite pattern emerged for goal-driven effort. These results demonstrate that how subjective measures of effort are framed (and interpreted by the respondent) can drastically influence how they relate to other variables of interest.

Effort inhabits a central location theoretically across a number of domains of inquiry. Nonetheless, the measurement of effort has long been fraught with difficulty. For example, while researchers have indexed effort using various measures including performance-based measures (e.g., encoding time, Koriat & Nussinson, 2009), physiological measures (e.g., pupil dilation, Beatty, 1982; systolic blood pressure, Gendolla et al., 2008), and subjective measures (e.g., self-report, Paas, 1993), these measures will often diverge from each other (Baars et al., 2020; DeLeeuw, 2008; Paas et al., 1994). Here we focus on one instance of such divergence within the context of subjective measures of effort. Namely, the manner in which subjective measures of effort are framed can have substantial effects on those effort reports and how those effort reports relate to other variables of theoretical interest (Koriat et al., 2014).

## Data-driven vs goal-driven effort

Subjective measures of effort require collecting a self-report from the participant. How this request is framed, of course, has the potential to impact how the individual interprets the request and as a result the answer provided. One important distinction is between the effort putatively required by a task and the effort an individual chooses to invest (Koriat, 2018; Koriat et al., 2006, 2009, 2014; Mulert et al., 2007). For example, Mulert et al. (2007) had participants distinguish between mental effort demand and volitional mental effort—characterising the former as passive (i.e., the effort associated with the task), and the latter as active (i.e., the effort individuals spend on the task). Participants made these self-reports following six different auditory tasks designed to vary in mental effort demand. These authors found a dissociation between mental effort demand and volitional mental effort such that reports of mental effort demand varied significantly between tasks; however, volitional mental effort remained stable (Mulert et al., 2007).

Koriat and colleagues (Koriat, 2018; Koriat et al., 2006, 2014; Koriat & Nussinson, 2009) examined a similar distinction between two different effort reports using data- and goal-driven effort frames. Koriat et al. (2014, p. 1625) defined data-driven effort as “the amount of effort required by the task in a bottom-up fashion” and goal-driven effort as “the amount of effort that the person wilfully invests in a task in a top-down fashion.” Across a series of studies, Koriat and colleagues (Koriat, 2018; Koriat et al., 2006, 2014; Koriat & Nussinson, 2009) have demonstrated that whether individuals interpret effort as data- or goal-driven can alter the relation between measures of effort and measures of metacognition and performance.

In the Experiment 1 by Koriat et al. (2014), effort framing was manipulated by assigning participants into two groups: a data-driven condition or a goal-driven condition. This manipulation involved both an instructional component and different frames for the self-reports of effort. With respect to the instructional component, participants were asked to read four stories during the practice phase and imagine that they were studying for a later exam. Participants in the data-driven condition were told that certain topics require more or less study effort, while those in the goal-driven condition were told that there are topics for which one chooses to invest more or less study effort. After each paragraph, participants completed effort self-reports; participants in the data-driven condition rated the amount of study effort the paragraph required, whereas participants in the goal-driven condition rated the amount of study effort they chose to invest in the paragraph. Subsequently, participants provided a judgement of learning (JOL) regarding their ability to accurately answer a question about the paragraph. Following this stage, participants completed a recall test containing one open-ended question for each paragraph. In the experimental phase of Experiment 1, participants completed the self-paced study of 60 word pairs and provided an effort report and JOL for each item, and following the study list completed a timed recall test. Koriat et al. (2014) found an interaction between effort ratings and item-level JOLs. When participants were provided a data-driven frame there was a clear negative association between effort and JOLs; as required effort reports increased, confidence in recall ability decreased. However, when participants were provided a goal-driven frame, there was a small positive association between effort and JOLs; as reports of wilfully invested effort increased, confidence in recall ability increased. Effort ratings interacted with performance on the recall test similarly to JOLs. For the data-driven condition, required effort was negatively associated with performance on the recall test; conversely, for the goal-driven condition, wilfully invested effort was weakly positively associated with performance on the recall test. Koriat et al. (2014) found similar results in Experiment 2, when participants watched an individual study for either a short or long duration, and then judged the individual’s likelihood of recalling a studied item and effort, using either a data-driven or goal-driven frame. That is, participants in the data-driven condition reported higher JOLs when the viewed individual studied an item for a “short” relative to a “long” duration; comparatively, participants in the goal-driven condition reported higher JOLs when the viewed individual studied an item for a “long” relative to a “short” duration. Baars et al. (2020) examined this complex relation between effort and metacognitive judgements in a meta-analysis (which included the research by Koriat and colleagues) and found that while the relations between effort measures and metacognitive judgements are in general negative (e.g., as effort, diversely measured, increases, JOLs decrease) they become less negative when a goal-driven frame (e.g., sense of agency) or manipulation (e.g., incentives) is used.

Theoretically, Koriat et al. (2014) proposed that the relation between effort and metacognitive judgements was the result of an attribution process that occurs post-study, in which participants interpret subjective effort in terms of a data- and goal-driven component. Specifically, they suggested that “ . . . learners partition the amount of study effort invested in each item into a data-driven component and a goal-driven component. The former component contributes towards reducing one’s JOLs, whereas the latter component contributes towards enhancing one’s JOLs” (see Koriat et al., 2014, p. 1625). A different conceptualisation might view each type of effort report as unique, and their relation determined by how individuals regulate effort. For example, one might choose to invest what is required. The distinction between data- and goal-driven effort has important implications for both the measurement of effort and our theoretical understanding of the construct (e.g., how it relates to other variables). Here, we further examine these ideas with a novel approach to examining the distinction between data- and goal-driven effort, an examination of how these different types of effort relate to affective variables, and a conceptual replication of the patterns reported in the literature.

## Present Investigation

In the present investigation, participants were tasked with learning content presented in the form of a video lecture. Previous studies on the distinctions between data- and goal-driven effort have focused on word and paired associate learning, and problem-solving tasks (for a review, see Baars et al., 2020). As noted above, Koriat and colleagues’ research comparing data- and goal-driven effort has typically had two different groups provided with the two distinct effort frames. While valuable, this kind of design leaves unanswered an important question about the relation between the two different effort reports. That is, in a given task how is data-driven effort related to goal-driven effort? To address this question, rather than have different groups led to interpret/report effort in either a data- or goal-driven fashion, we had the same participants provide both data- and goal-driven effort reports. This enabled us to examine the relation between data- and goal-driven effort reports. While Mulert et al. (2007) also collected their “mental effort demand” and “volitional mental effort” ratings from each participant they did not report the relation between the two. As noted above, one conceptualisation of their relation could be derived from the work by Koriat et al. (2014)—if individuals partition a fixed amount of effort into a data- and goal-driven component, then these two effort reports should be negatively correlated. That is, given a fixed amount of effort, if data-driven effort increases, then goal-driven effort has to decrease (and vice versa). A different conceptualisation of the two effort reports is that individuals treat them as two distinct judgements. For example, judgements of how much effort a cognitive activity required might be more centred on the interaction between characteristics of the individual and task, whereas judgements of how much effort one chose to invest might be viewed as a kind of direct report of their resource allocation to the task. On this account, there is no theoretical requirement that a relation exists between these two judgements. That said, one might intuit that data-driven effort reports would be positively correlated with goal-driven effort reports as they are likely both related to an individual’s processes of volitional regulation (Corno & Kanfer, 1993); thus, in many contexts how much effort a task requires will be related to how much effort an individual chooses to invest. This would predict a positive relation between data- and goal-driven effort.

In addition to examining the bivariate relation between data- and goal-driven efforts, collecting both of these effort reports also affords a unique opportunity to statistically examine their (potential) differential relations with other variables. That is, we can ask how data-driven effort relates to a given variable of theoretical interest while controlling for goal-driven effort, and how goal-driven effort relates to a given variable of theoretical interest while controlling for data-driven effort. This type of approach is particularly valuable if, as suggested above, these two effort reports are correlated. In addition, if, as suggested by Koriat and colleagues (Koriat, 2018; Koriat et al., 2006, 2014; Koriat & Nussinson, 2009), these two types of efforts have opposing relations to a given variable, then statistically controlling for one while assessing the other could more clearly reveal the unique associations present. For example, if, as suggested above, individuals invest the amount of the effort that a task requires of them, then controlling for the influence of data-driven effort will allow us to examine the independent association between goal-driven effort and our variables of interest. Thus, after viewing the lecture, participants provided both a data-driven effort (i.e., “how much mental effort did the lecture require?”) and a goal-driven effort (i.e., “how much mental effort did you choose to invest while watching the lecture?”) report, in addition to other measures (described below), and finally completed a recall test based on the lecture material as a measure of memory. To examine the relation between data- and goal-driven effort with metacognition, we collected two types of metacognitive judgements; a prospective performance estimate (i.e., a prediction about how well the participant thought they would do on the upcoming recall test) and a retrospective performance estimate (i.e., a prediction about how well they did on the recall test after completing it). Based on the work by Koriat and colleagues (2006, 2009, 2014, 2018), we expect data-driven effort reports to be negatively correlated with prospective and retrospective performance estimates and with test performance; on the contrary, we expect goal-driven effort reports to be positively correlated with prospective and retrospective performance estimates and with test performance, or at least less negatively correlated (see Baars et al., 2020). Confirming our predictions would generalise Koriat and colleagues’ findings to a novel learning task and method of assessing data- and goal-driven effort.

In addition to examining the relations between data- and goal-driven effort, with metacognition and performance, we sought to extend extant research on these different effort reports to include affective variables. Specifically, we examined subjective reports of positive and negative affect, one’s liking of the lecture, and one’s willingness to watch a similar lecture in the future. Effort is typically considered aversive and avoided if possible (Dunn et al., 2019; Dunn & Risko, 2019; Kool et al., 2010; Kurzban, 2016). Thus, a simple prediction might be that there would exist a negative relation between both data- and goal-driven effort and affect. However, the relation between effort and affect is arguably more complex (see Inzlicht et al., 2018) and importantly for this work, might map onto data- and goal-driven effort differently. Inzlicht et al. (2018) discussed a number of scenarios where effort is associated with positive emotions. For example, when people are exerting effort in a state of flow, they experience emotions such as enjoyment and engagement. Inzlicht et al. (2018) also identified a number of cases where individuals seem to like engaging in effortful tasks. For example, some marathoners value running because it is effortful and as such, experience effort as a secondary reinforcer. Returning to data- and goal-driven effort, the wording of the data- and goal-driven effort measures (i.e., “require” vs “choose”) might activate notions related to the absence/presence of autonomy. Autonomy is generally preferred (Bown et al., 2003; Flowerday & Schraw, 2003; Spector, 1986), thus increases in the effort “required” might be perceived negatively, whereas increases in effort arising from a choice might be perceived less negatively or even positively. In a similar vein, framing effort investment as a choice (i.e., goal-driven) might lead individuals to perceive it more positively to avoid potential dissonance arising from reporting that you chose to engage in an act that you perceived negatively (Aronson & Mills, 1959; Brehm, 1956; Kim & Song, 2020).

## Methods

### Participants

Data were collected from 207 participants (127 women, 78 men, and 2 others) living in Canada, the United States, and the United Kingdom from an online recruiting platform, Prolific. Twenty-two participants were excluded from further analyses because they did one or more of the following: used the internet to search for test answers, took notes during the video lecture, did not watch the full video lecture, indicated that we should not use their data (e.g., difficulty encoding verbal information presented in the video lecture), or failed an attention check during the test. Moreover, data from three participants were excluded because they reported a technical glitch that led them to rewatch the first video segment.

The sample size was based on unpublished studies in the laboratory examining relations between effort reports and other variables. The final sample size of 182 gives us 80% power, at a significance criterion of α = .05, to detect small-sized correlations (*r* = .2).

### Materials

#### Demographics

Participants were asked to provide their age, gender, and the number of online courses they had taken. Participants were also asked to indicate their education level by selecting one of the following: 1 = “*primary*/*elementary schooling*,” 2 = “*high school diploma*,” 3 = “*college diploma*,” 4 = “*bachelors degree*,” 5 = “*masters degree*,” 6 = “*doctorate degree*,” and 7 = “*no formal education*/*other*.” Moreover, participants were asked to indicate their level of proficiency in reading and listening in English on 4-point self-report scales, ranging from 1 (*no proficiency*) to 4 (*high proficiency*).

#### Lecture

Participants viewed an 11-min introduction to statistics video lecture (Szpunar et al., 2013). The video lecture content provided an overview of how statistical studies are used to infer information about the population. The video lecture displayed presentation slides paired with audio from the professor during the class. Participants were instructed to attend to the lecture and not take notes. The video lecture was split into two 5.5-min segments between which there was a 30-s break.

#### Effort

Participants’ perceptions of data- and goal-driven effort were measured using two 9-point self-report scales from 1 (*very, very little*) to 9 (*very, very much*). For data-driven effort, participants rated the amount of effort required by the video lecture (i.e., “how much mental effort did the lecture require?”). For goal-driven effort, participants rated the amount of effort they chose to invest into the video lecture (i.e., “how much mental effort did you choose to invest while watching the lecture?”).

#### Test

Memory for lecture material was measured using performance on a recall test. The test contained 12 questions from the video lecture, which were either short answer or fill-in-the-blank format.

#### Metacognitive judgements

Participants estimated how well they thought they would perform on the recall test (prospective) and estimated how well they did perform on the recall test (retrospective). These items were answered using a percentage selected from a sliding scale that ranged from 0 to 100.

#### Affect

##### Affective experiences

Participants’ affective experience during the video lecture was measured using the Positive and Negative Affect Schedule (PANAS; Watson et al., 1988). The PANAS is a 20-item positive and negative affect assessment questionnaire consisting of two subscales: positive affect (e.g., “*excited*,” “*alert*,” “*inspired*”) and negative affect (e.g., “*distressed*,” “*upset*,” “*irritable*”; Watson et al., 1988). This scale allows the computation of a positive affect score and a negative affect score. Participants rated how often they experienced positive and negative affect while watching the video lecture (i.e., “how much you experienced any of these feelings while watching the video lecture”) on a five-point scale from 1 (*not at all*) to 5 (*a lot*).

##### Affective evaluations

Participants’ affective evaluation of the lecture was measured using two 7-point self-report scales. The liking scale measured participants’ liking of the lecture task by reporting how much they liked the video lecture (i.e., “how much you liked the video lecture you just watched”), ranging from 1 (*very much disliked*) to 7 (*very much liked*). The future consumption scale asked participants how likely they would be to watch another video lecture presented in a similar format (i.e., “how likely it is you would watch another video lecture that was presented to you in this format”), ranging from 1 (*very unlikely*) to 7 (*very likely*).

### Procedure

This study received ethics clearance through a University of Waterloo Research Ethics Board. Participants signed up for the study on Prolific and opened a link to a Qualtrics survey. The Qualtrics survey began with two reasonably easy questions designed to increase data quality by preventing bot responses. If both of these questions were answered correctly, then the information letter and online consent form were presented. After informed consent was given, participants started the study by completing the demographic questions. Participants were instructed that the video lecture would be split into two segments between which there would be a short break. Participants were asked to attend to the video lecture, not take notes during the video lecture, to ensure their audio was working, and when ready click to the next page—at which time the video lecture would automatically begin. The first video segment was followed by a screen informing the participant that there would be a 30-s break after which the second video segment would automatically begin. After watching the video lecture, participants completed the effort and affect self-report measures. The order of presentation for these variables was counterbalanced (i.e., participants completed these measures in one of six possible orders) and the presentation of items within each measure was randomised. Items within the effort and affective evaluation measures were presented sequentially on different screens. Participants were then asked the topic of the video lecture and indicated their prospective performance estimate. Next, participants completed the test—presentation of test questions was randomised. After the test, participants indicated their retrospective performance estimate. Finally, participants answered four post-survey questions, to ensure that they followed study instructions correctly, and were then presented with a feedback letter.

### Data quality

Data quality measures were implemented to identify participants who were not following study instructions. These measures included attention checks and post-survey questions. Attention checks were placed throughout the study: after the video lecture participants had to report the topic, within the PANAS an item asked participants to select a particular option (i.e., “Pick option four”), and in the recall test participants had to select the larger number (i.e., “Which number is larger, 9 or 34?”). Finally, post-survey participants were told to honestly answer yes/no questions about whether they used Google to search for test answers, took notes during the video lecture, watched the video lecture fully, and whether we should use their data. Data from participants who failed an attention check and/or indicated on the post-survey questions that they did not adhere to the study instructions or that their data should not be used were removed from our analyses.

## Results

These analyses were pre-registered (https://doi.org/10.17605/OSF.IO/RQW75) on the Open Science Framework—predictions were not included in the pre-registration. Moreover, the data and associated R code are available on Open Science Framework (https://doi.org/10.17605/OSF.IO/PEQYD). Analyses presented below are based on the final data set of 182 participants (112 females, 69 males, and 1 other). The final sample (Mean age = 33.37) comprised data from highly educated participants—with 1% completing Primary/Elementary schooling, 19 % completing a High School diploma, 19% completing a College diploma, 42% completing a Bachelors degree, 17% completing a Masters degree, 2% completing a Doctorate degree, and 1% completing other/no formal education as their highest level of education. Moreover, the final sample reported a high level of proficiency in both listening (2% reported low proficiency, 12% reported moderate proficiency, and 86% reported high proficiency) and reading (14% reported moderate proficiency and 86% reported high proficiency) English. On average participants completed six online courses with 83% of participants completing at least one online course. The associations between these demographics and our dependent variables are discussed in the online Supplemental materials (see Appendix A).

There were moderate to severe violations of normality with a number of variables, particularly negative PANAS scores which were strongly skewed as individuals rarely experienced negative affect in our lecture. While inferences drawn from Pearson’s correlations are typically robust to such violations (see Puth et al., 2014, 2015), particularly with large sample sizes, we conducted parallel analyses using the nonparametric tests Spearman’s rho and Kendall’s tau. Similar patterns of results were obtained, Pearson’s correlations are provided below. Table 1 presents the bivariate correlations between each of our variables. Our focus is on the relation between effort and the other variables, but all the relations are presented for the interested reader. Table 2 presents the partial correlations between each effort report and the other variables controlling for the other effort report.

**Table 1.** Means (*M*), standard deviations (*SD*), and bivariate correlations between variables.

Variable	*M*	*SD*	1	2	3	4	5	6	7	8
1. Data-driven effort	6.17	1.84								
2. Goal-driven effort	6.82	1.55	.35**							
3. Test performance	0.58	0.23	–.35**	–.03						
4. Prospective estimate	0.62	0.21	–.41**	.13	.42**					
5. Retrospective estimate	0.52	0.25	–.35**	.02	.64**	.71**				
6. Positive affect	2.66	0.85	.13	.51**	.01	.30**	.20**			
7. Negative affect	1.49	0.49	.28**	–.01	–.21**	–.32**	–.21**	.00		
8. Liking	4.68	1.45	–.11	.36**	.13	.47**	.31**	.63**	–.20**	
9. Future consumption	4.45	1.72	–.01	.39**	.16*	.39**	.28**	.64**	–.14	.76**

The scale ranges are 1 to 9 for data- and goal-driven effort judgements; 0 to 1 for test performance, prospective estimates, and retrospective estimates; 1 to 5 for positive affect and negative affect; 1 to 7 for liking and future consumption.

* *p* < .05. **p < .01.

**Table 2.** Partial correlations between each type of effort report and test performance, metacognitive, and affect variables, controlling for the other type of effort report.

Variable	Data-driven effort	Goal-driven effort
Test performance	–.36**	.10
Prospective estimate	–.49**	.32**
Retrospective estimate	–.38**	.16*
Positive affect	–.06	.50**
Negative affect	.30**	–.12
Liking	–.27**	.43**
Future consumption	–.17*	.42**

* *p* < .05. **p < .01.

In addition, given the order of the effort and affect variables were counterbalanced, we also conducted bivariate and partial correlations separately for the subset in which participants completed the effort items first (*N* = 73) and the subset in which participants completed all of the affect items first (*N* = 55).^1^ Overall, the patterns of results within each subset were consistent with those observed in the full data set (see Appendix B in the online Supplemental materials). We only discuss discrepant results wherein a pattern qualitatively changes across orders (i.e., changes direction). We generally ignore changes in magnitude or *p*-values given the significant reduction in sample size associated with these analyses.

### Effort

Data- and goal-driven effort were positively correlated with each other at the bivariate level, *r*(180) = .35, *p* < .001.

### Test

At the bivariate level, data-driven effort was negatively correlated with test performance, whereas goal-driven effort was not correlated with test performance, *r*(180) = –.34, *p* < .001; *r*(180) = –.03, *p* = .69, respectively. When controlling for goal-driven effort, data-driven effort remained negatively correlated with test performance, *r*(179) = –.36, *p* < .001. When controlling for data-driven effort, goal-driven effort remained not correlated with test performance, *r*(179) = .10, *p* = .17.

### Metacognitive judgements

At the bivariate level, prospective and retrospective performance estimates were positively correlated with each other, *r*(180) = .71, *p* < .001, and with test performance, *r*(180) = .42, *p* < .001; *r*(180) = .64, *p* < .001, respectively.

At the bivariate level, data-driven effort was negatively correlated with prospective and retrospective performance estimates, *r*(180) = –.41, *p* < .001; *r*(180) = –.35, *p* < .001, respectively; whereas, goal-driven effort was not correlated with prospective or retrospective performance estimates, *r*(180) = .13, *p* = .09; *r*(180) = .02, *p* *=* .82, respectively. When controlling for goal-driven effort, data-driven effort remained negatively correlated with prospective and retrospective performance estimates, *r*(179) = –.49, *p* < .001; *r*(179) = –.38, *p* < .001, respectively. When controlling for data-driven effort, goal-driven effort was positively correlated with prospective and retrospective performance estimates, *r*(179) = .32, *p* < .001; *r*(179) = .16, *p* = .03, respectively.

### Affect

At the bivariate level, data-driven effort was not correlated with positive affect, *r*(180) = .13, *p* = .08, but was positively correlated with negative affect, *r*(180) = .28, *p* < .001; whereas, goal-driven effort was positively correlated with positive affect, *r*(180) = .51, *p* < .001, but was not correlated with negative affect, *r*(180) = –.01, *p* = .88. Partial correlations revealed similar findings. When controlling for goal-driven effort, data-driven effort remained not correlated with positive affect, *r*(179) = –.06, *p* = .43, and positively correlated with negative affect, *r*(179) = .31, *p* < .001.^2^ When controlling for data-driven effort, goal-driven effort remained positively correlated with positive affect, *r*(179) = .50, *p* < .001, and not correlated with negative affect, *r*(179) = –.12, *p* = .10.

At the bivariate level, data-driven effort was not correlated with liking or future consumption, *r*(180) = –.11, *p* = .14; *r*(180) = –.01, *p* = .95, respectively; whereas, goal-driven effort was positively correlated with both of these variables, *r*(180) = .36, *p* < .001; *r*(180) = .39, *p* < .001, respectively. When controlling for goal-driven effort, data-driven effort was negatively correlated with liking and future consumption, *r*(179) = –.27, *p* < .001; *r*(179) = –.17, *p* = .03, respectively. When controlling for data-driven effort, goal-driven effort remained positively correlated with liking and future consumption, *r*(179) = .43, *p* < .001; *r*(179) = .42, *p* < .001, respectively.

At the bivariate level, positive affect was positively correlated with both liking and future consumption, *r*(180) = –.63, *p* < .001; *r*(180) = .64, *p* < .001, respectively; whereas, negative affect was negatively correlated with liking and marginally negatively correlated with future consumption, *r*(180) = –.20, *p* = .01; *r*(180) = –.14, *p* = .05, respectively.

## Discussion

This study examined the relation between data- and goal-driven effort reports and their relation to performance, metacognition, and affect both independently and when controlling for the contribution of the other type of effort report. Data- and goal-driven effort reports were moderately positively correlated. At the bivariate level, data-driven effort reports were moderately negatively correlated with performance, metacognition, and affect; a similar pattern emerged when the contribution of goal-driven effort reports was controlled. At the bivariate level, goal-driven effort reports were moderately positively correlated with affect; when controlling for the contribution of data-driven effort, a positive correlation with metacognitive judgements also emerged. Clearly, the manner in which subjective effort reports are framed can have substantial impacts on how those reports are related to other variables.

Koriat et al. (2014) proposed that learners partition effort into a data- and goal-driven component. This conceptualisation suggests that data- and goal-driven effort reports would be negatively correlated. The present results are inconsistent with the view by Koriat et al. (2014). Data- and goal-driven effort were moderately positively correlated. As reports of effort required by the lecture increased, so did reports of effort one chose to invest in the lecture. This finding is consistent with the view proposed in the introductory paragraph that these effort reports are both related to an individual’s processes of volitional regulation (Corno & Kanfer, 1993). In the introductory paragraph, we suggested that individuals might treat these data- and goal-driven questions as two different judgements. On this account, there is no theoretical requirement that there will be a correlation but, of course, there may well be depending on how individuals make these respective judgements. One approach on this front would be to propose that each effort judgement is a kind of inference based on available cues (Ashburner & Risko, 2021, 2022; Dunn et al., 2016; Dunn & Risko, 2019) similar to other metacognitive judgements (Koriat, 1997; Koriat & Levy-Sadot, 2001; Metcalfe et al., 1993). Furthermore, given the clear differences between data- and goal-driven effort that we and others have observed, one could further argue that the cues utilised, at least to some extent may differ across judgements (see Scheiter et al., 2020). For example, the topic of the lecture here was statistics and individuals might have pre-experimental beliefs about how effortful it is to learn statistical concepts in general or for them personally. This kind of topic-related belief might inform perceptions of data-driven effort more so than goal-driven effort. Goal-driven effort, on the contrary, might be more influenced by the experienced intensity of resource allocation. For example, participants might recall actively inhibiting engaging in secondary tasks (e.g., media-multitasking, Ralph et al., 2020; mind wandering, Risko et al., 2012) and/or actively engaging in cognitive activities associated with deeper learning (e.g., germane processing, Sweller et al., 2019). The inference-based approach articulated here draws attention to the need to understand more deeply the information individuals use (or do not use) in effort reports and effort-based decisions more broadly. Future research, examining how person level variables (e.g., previous knowledge, interest) relate to data-driven and goal-driven effort, which we did not collect here, would represent a particularly valuable direction.

### Performance, metacognition, and effort

With respect to the relation between data- and goal-driven effort reports with performance and metacognitive judgements, the results were generally consistent with the previous demonstrations by Koriat and colleagues’ (2006, 2009, 2014, 2018) and the meta-analysis by Baars et al. (2020). In both bivariate and partial correlation analyses, data-driven effort was moderately negatively correlated with prospective and retrospective performance estimates, and with test performance. As perceptions of required effort increased, individuals predicted a decrease in performance and performed worse; even when controlling for how much effort one chose to invest. Koriat et al. (2006) suggested that the negative associations between data-driven effort with performance and metacognitive judgements might be driven by fluency, the ease at which information is encoded or retrieved (see also Reber & Greifeneder, 2017). From this perspective, metacognitive experiences associated with disfluent information processing during the video lecture may have led to higher appraisals of required effort and lower memory performance.

Concerning goal-driven effort, the predicted positive associations (based on past work) with performance estimates (prospective and retrospective) and test performance were not observed at the bivariate level; though a moderate positive correlation with prospective, and a weak positive correlation with retrospective, performance estimates emerged in the partial correlation analyses. Controlling for required effort, as reports of wilfully invested effort increased, individuals’ performance estimates increased, though their actual test performance was unrelated. Koriat et al. (2006) suggested that the positive association between goal-driven effort and metacognitive judgements might be explained by the extent to which individuals engaged strategic efforts towards learning (e.g., trying to connect information learnt across the lecture or to pre-experimental knowledge). From this perspective, the extent to which an individual engaged in such learning strategies together with an assumption that such strategies are effective would lead to higher predictions and higher reports of wilfully invested effort. The failure to find a relation between goal-driven effort and actual performance might reflect the relative ineffectiveness of those efforts. That is, the prediction that goal-driven effort will be positively correlated with test performance assumes that effort invested in learning from the video lecture will improve performance. This need not be the case. For example, if participants lack the capacity to meet the demands of the task in the moment, then increasing effort might be superfluous. In a similar vein, tasks that are too easy would benefit little from investing effort. The potential complexity of the relation between goal-driven effort and performance also highlights the importance of considering assumptions about the linear relations between effort reports and other variables of interest. For example, the relation between goal-driven effort and performance might be low for easy tasks, higher for intermediate difficulty tasks, and low again for extremely difficult tasks. Furthermore, in exploring the correlations between effort reports and performance (and other variables examined here as well), it needs to be acknowledged that the direction of causation cannot be clearly established.

### Affect and effort

This investigation revealed a clear dissociation between data- and goal-driven effort reports and affect. Specifically, data-driven effort seems to be negatively valenced while goal-driven effort seems to be positively valenced. Affect was operationalised here in terms of affective experiences and affective evaluations, but the pattern of relations with effort reports was similar across these affective measures.

Effort has long been considered a cost to be avoided (Dunn et al., 2019; Dunn & Risko, 2019; Kool et al., 2010; Kurzban, 2016). This might reflect the opportunity cost associated with tying up limited resources (Kurzban et al., 2013). The dissociation between data- and goal-driven effort with affect observed here suggests a novel perspective on this idea. That is, (increases in) effort might be more likely to be interpreted as a cost when its expenditure is associated with a lack of autonomy, it is “required” (data-driven effort); and more positively when its expenditure is associated with control, it is “given” (goal-driven effort). As noted in the introductory paragraph, a lack of autonomy is generally viewed negatively relative to being in control (Bown et al., 2003; Haase et al., 2012; Pekrun, 2006; Spector, 1986). In addition, framing goal-driven effort as a choice might prompt positive evaluations to avoid dissonance (Bem, 1967; Klein et al., 2005). Thus, the sole act of tying up limited resources might not be experienced as negative. Instead, the experience of autonomy associated with the act of tying up those limited resources may determine the resulting affective response.

Increases in data-driven effort might also be related to a threat of failure and the latter to negative affect like frustration and anxiety (deMarrais & Tisdale, 2002; Elliott, 1988; Pekrun, 2006). As the requirements of a task increase, the likelihood that an individual cannot mobilise sufficient resources to succeed (e.g., understand the lecture material enough to do well on the test) would also increase. This pattern of association would be less likely in the context of goal-driven effort given the decision is framed as autonomous (i.e., one could not invest more than one has to invest). Of course, there may be a number of different contributions to the affective quality of data- and goal-driven effort. Future research aimed specifically at understanding this dissociation would be valuable.

In at least one case, the presentation order of the effort and affect items affected the relations between them significantly. Specifically, when effort was presented before affect, there was a moderately positive (and significant) association between data-driven effort and positive PANAS but when affect was presented before effort there was a similar magnitude (but not significant) negative association between these same variables. Thus, the null bivariate association between data-driven effort and positive affect appears to reflect two opposing correlations as a function of order. While the sample sizes were modest, this pattern suggests that in future research the order of effort and other reports needs to be carefully considered.

### Methodological implications

The moderate positive correlation between data- and goal-driven effort suggests that considering either type of effort independently of the other may lead to misinterpretations of the relations between these effort reports and other variables of theoretical interest. The contrast between the bivariate and partial correlation analyses observed here further supports this idea. That is, oftentimes the relations between data- and goal-driven effort with other variables at the bivariate level were different from those revealed when those relations were assessed controlling for the other type of effort report. For example, at the bivariate level, goal-driven effort was unrelated to prospective and retrospective performance estimates but was moderately positively correlated to both when controlling for data-driven effort. Similarly, at the bivariate level, data-driven effort was unrelated to both liking and future consumption but was weakly negatively correlated to both when controlling for goal-driven effort.

It is important to note that, at least in this case, the correlations at the bivariate level were generally directionally consistent with those observed when controlling for the other type of effort report. Furthermore, data- and goal-driven effort reports were associated with the target variable in the opposite direction in the partial correlation analyses. This suggests that at the bivariate level the positive relation between data- and goal-driven effort may be suppressing the unique association between each effort report and the variables of interest where differences between the bivariate and partial correlations were found. For example, revealing the positive relation between goal-driven effort and performance estimates (e.g., “if I invest more effort, then I will perform better”) may require controlling for data-driven effort because the latter is negatively associated with performance estimates (e.g., “the more effort a task requires, then the less well I will be able to perform”).

### Difficulty, data-driven, and goal-driven effort

This investigation has contrasted data-driven effort, the amount of effort a task required, and goal-driven effort, the amount of effort one chose to invest in a task. Both of these constructs are, of course, closely related to subjective difficulty. Indeed, Paas et al. (1994) conceptualise difficulty as a causal factor that influences cognitive load and effort as an assessment factor affected by cognitive load. These authors consider difficulty to be a more task-centred dimension, focused on characteristics of the task environment, and effort as a more human-centred dimension, centred on the cognitive resources invested in a task. The latter seems to clearly align with the conceptualisation of goal-driven effort used here and by Koriat and colleagues (2006, 2014). Hence, data-driven effort might align better with difficulty; nonetheless, there may be important distinctions worth making. Koriat and colleagues (2006, 2014) suggested that data-driven effort is determined by the learner-item interaction (e.g., “how much effort did the task require of me”). One can imagine answering a difficulty question in the same manner (e.g., “how difficult was the task for me”). However, one might also report on how much effort a task required, or how difficult a task was, with reference to task characteristics apropos to some generic cognitive agent (e.g., “how much effort would the task require of people in general”; “how difficult would the task be for people in general”). Consider the task of driving, when first learning how to drive the task is perceived as requiring a great amount of effort, or as difficult, but with practice, driving is perceived as requiring little effort or being less difficult. This conceptualisation of data-driven effort and difficulty centres around characteristics of the individual–task interaction. The task of driving itself does not change, rather the person changes (i.e., they learn how to operate the automobile). From this perspective, one could, in principle, construct a data-driven effort or difficulty measure that instead drew greater attention to the task and less to the interaction in an attempt to assess individuals’ beliefs about the general relation between task characteristics and data-driven effort/difficulty. Thus, in considering the distinction between data-driven effort and difficulty, it would be valuable to understand how respondents interpret the terms. In the framework developed above, one might frame the question as an examination of the cues that are primed when the different frames are used (e.g., goal-driven effort vs data-driven effort vs difficulty). In this study context, the wording of the data-driven effort item clearly implies an effort report that considers the interaction between the respondent and the task. That is, it was posed after completion of a task and asked how much effort “did” the task require.

## Conclusion

Overall, these findings demonstrate that data- and goal-driven effort reports are differentially related to performance, metacognition, and affect. The approach adopted here provided a new perspective on how relations between these effort reports and other variables can be approached. Future research using both data- and goal-driven effort reports will provide a deeper understanding of the perception of effort.

## Supplemental Material

sj-docx-1-qjp-10.1177_17470218231186609 – Supplemental material for Dissociations between data-driven and goal-driven effort reports: Performance, metacognition, and affect Supplemental material, sj-docx-1-qjp-10.1177_17470218231186609 for Dissociations between data-driven and goal-driven effort reports: Performance, metacognition, and affect by Kate Van Kessel, Michelle Ashburner and Evan F Risko in Quarterly Journal of Experimental Psychology

## Notes

1. The subset in which participants did not complete affect items in succession (*N* = 54) was not examined in our order analyses.

2. Interestingly, controlling for goal-driven effort, when effort was presented before affect, data-driven effort was positively correlated with positive affect, *r*(70) = .25, *p* = .03; but when affect was presented before effort, data-driven effort was not significantly correlated with positive affect, though the correlation trended in a negative direction, *r*(52) = –.21, *p* = .12. Interpretation of the latter should take into account the increased likelihood of a type 2 error given that the sample size was not sufficiently powered. The association between data-driven effort and positive affect represents the most divergent pattern observed in the order analyses and is discussed further in the general discussion.
