# Supplementary material for: Dissociations between data-driven and goal-driven effort reports: Performance, metacognition, and affect
Source: Q J Exp Psychol (Hove). 2023 Jul 31;77(5):983–93. doi: 10.1177/17470218231186609 (PMC11032630; doi:10.1177/17470218231186609)
Supplement: sj-docx-1-qjp-10.1177_17470218231186609 – Supplemental material for Dissociations between data-driven and goal-driven effort reports: Performance, metacognition, and affect [file sj-docx-1-qjp-10.1177_17470218231186609.docx]

Supplemental Material for:

**Dissociations between Data-Driven and Goal-Driven Effort Reports: Performance, Metacognition, and Affect**

Kate Van Kessel, Michelle Ashburner, Evan F. Risko

Department of Psychology, University of Waterloo

***Corresponding author:***

Van Kessel Kate

University of Waterloo Faculty of Arts - Psychology

Waterloo, Ontario

Canada

https://orcid.org/0000-0002-4677-8008

[krvankes@uwaterloo.ca](mailto:krvankes@uwaterloo.ca)

**Appendix A**

**Associations with Demographics**

As stated in our pre-registration (https://osf.io/rqw75) we report the associations between our demographic variables and our dependent variables of interest. Gender had one individual in the other category and as such the variable was treated as dichotomous using only male and female. English proficiency, both reading and listening, had the large majority (n = 157) fall in the high proficiency category and the remainder (n = 25) in the low and moderate proficiency category. As such, we also dichotomized this variable into high and low proficiency. T-tests were conducted to examine the associations between these variables with our dependent variables of interest. Descriptive data for these variables are presented in Table S1. Age, education level, and number of online courses were treated as continuous variables in a correlational analysis. Pearson’s correlations between these demographic variables and our dependent variables of interest are reported below (see Table S2). Following the procedure used in the main text, parallel analyses using the nonparametric tests Spearman's rho and Kendall's tau were conducted and qualitatively similar patters of results were obtained.

***Gender***

Welch Two Sample *t*-tests were used to examine differences between males and females. Overall, no significant differences were observed (all *p*s > .10) with the exception of one variable, retrospective performance estimates. Male participants (*M* = 0.58, *SD* = 0.24) provided higher retrospective performance estimates compared to female participants (*M* = 0.48, *SD* = 0.26), *t*(154.59) = 2.42, *p* = .02.

***English Proficiency***

Welch Two Sample *t*-tests were used to examine potential differences between participants’ English proficiency levels. For both English reading and listening proficiency, participants who reported high proficiency (*M* = 0.60, *SD* = 0.21; *M* = 0.60, *SD* = 0.21) had higher test performance compared to those who reported lower proficiency (*M* = 0.44, *SD* = 0.28; *M* = 0.43, *SD* = 0.27), *t*(28.48) = 2.83, *p* = .008; *t*(28.88) = 3.06, *p* = .005. For both English reading and listening proficiency, participants who reported high proficiency (*M* = 1.43, *SD* = 0.41; *M* = 1.43, *SD* = 0.41) had lower reports of negative affect compared to those who reported lower proficiency (*M* = 1.81, *SD* = 0.76; *M* = 1.82, *SD* = 0.76), *t*(26.28) = -2.44, *p* = .02; *t*(26.27) = -2.53, *p* = .02. For English reading proficiency, participants who reported high proficiency (*M* = 0.54, *SD* = 0.24) provided higher retrospective performance estimates compared to those who reported lower proficiency (*M* = 0.39, *SD* = 0.32), *t*(28.41) = 2.13, *p* = .04. No other significant differences were observed (all *p*s > .06).

**Table S1**

*Means (M), standard deviations (SD) of variables as a function of gender and English proficiency.*

| Variable | Gender | | | |  | Reading Proficiency | | | |  | Listening Proficiency | | | |
| --- | --- | --- | --- | --- | --- | --- | --- | --- | --- | --- | --- | --- | --- | --- |
|  | Male | | Female | |  | Low | | High | |  | Low | | High | |
|  | *M* | *SD* | *M* | *SD* |  | *M* | *SD* | *M* | *SD* |  | *M* | *SD* | *M* | *SD* |
| 1. Data-Driven Effort | 5.87 | 2.01 | 6.35 | 1.73 |  | 6.84 | 2.03 | 6.06 | 1.80 |  | 6.68 | 1.99 | 6.09 | 1.81 |
| 2. Goal-Driven Effort | 6.58 | 1.85 | 6.99 | 1.31 |  | 7.04 | 1.65 | 6.79 | 1.54 |  | 7.20 | 1.32 | 6.76 | 1.58 |
| 3. Test Performance | 0.57 | 0.22 | 0.59 | 0.23 |  | 0.44 | 0.28 | 0.60 | 0.21 |  | 0.43 | 0.27 | 0.60 | 0.21 |
| 4. Prospective Estimate | 0.65 | 0.20 | 0.60 | 0.21 |  | 0.54 | 0.24 | 0.63 | 0.20 |  | 0.54 | 0.24 | 0.63 | 0.20 |
| 5. Retrospective Estimate | 0.58 | 0.24 | 0.48 | 0.26 |  | 0.39 | 0.32 | 0.54 | 0.24 |  | 0.40 | 0.33 | 0.54 | 0.24 |
| 6. Positive Affect | 2.76 | 0.83 | 2.60 | 0.86 |  | 2.47 | 0.84 | 2.69 | 0.85 |  | 2.44 | 0.82 | 2.69 | 0.85 |
| 7. Negative Affect | 1.52 | 0.59 | 1.47 | 0.42 |  | 1.81 | 0.76 | 1.43 | 0.41 |  | 1.82 | 0.76 | 1.43 | 0.41 |
| 8. Liking | 4.78 | 1.36 | 4.63 | 1.51 |  | 4.68 | 1.41 | 4.68 | 1.46 |  | 4.72 | 1.46 | 4.68 | 1.46 |
| 9. Future Consumption | 4.49 | 1.59 | 4.44 | 1.80 |  | 4.44 | 1.89 | 4.45 | 1.69 |  | 4.48 | 1.90 | 4.45 | 1.69 |

***Age***

Age was negatively correlated with test performance such that as age increased test performance decreased, *r*(180) = -.18, *p* = .01. Age was also positively correlated with positive affect such that as age increased reports of positive affect increased, *r*(180) = .21, *p* = .005. No other significant correlations were observed (all *p*s > .06).

***Education Level***

Education level was negatively correlated with data-driven effort such that as education level increased reports of effort required by the lecture task decreased, *r*(180) = -.19, *p* = .01. Education level was also positively correlated with test performance and both prospective and retrospective performance estimates such that as education level increased test performance and performance estimates also increased, *r*(180) *=* .21, *p =* .004; *r*(180) = .16, *p =* .03; *r*(180) = .19, *p* = .001, respectively. Lastly, education level was negatively correlated with negative affect such that as education level increased reports of negative affect decreased, *r*(180) = -.24, *p* = .001. No other significant differences were observed (all *p*s > .09).

***Online Courses Taken***

There were no significant correlations between the number of online courses taken and any of our dependent variables of interest (all *p*s > .13).

*.*

**Table S2**

*Means (M), standard deviations (SD), and bivariate correlations between variables and demographics*

| Variable | *M* | | *SD* | 1 | 2 | 3 | 4 | 5 | 6 | 7 | 8 | 9 | 10 | 11 |
| --- | --- | --- | --- | --- | --- | --- | --- | --- | --- | --- | --- | --- | --- | --- |
| 1. Data-Driven Effort | 6.17 | 1.84 | |  |  |  |  |  |  |  |  |  |  |  |
| 2. Goal-Driven Effort | 6.82 | 1.55 | | .35** | |  |  |  |  |  |  |  |  |  |
| 3. Test Performance | 0.58 | 0.23 | | -.35** | -.03 |  |  |  |  |  |  |  |  |  |
| 4. Prospective Estimate | 0.62 | 0.21 | | -.41** | .13 | .42** | |  |  |  |  |  |  |  |
| 5. Retrospective Estimate | 0.52 | 0.25 | | -.35** | .02 | .64** | .71** | |  |  |  |  |  |  |
| 6. Positive Affect | 2.66 | 0.85 | | .13 | .51** | .01 | .30** | .20** | |  |  |  |  |  |
| 7. Negative Affect | 1.49 | 0.49 | | .28** | -.01 | -.21** | -.32** | -.21** | .00 |  |  |  |  |  |
| 8. Liking | 4.68 | 1.45 | | -.11 | .36** | .13 | .47** | .31** | .63** | -.20** | |  |  |  |
| 9. Future Consumption | 4.45 | 1.72 | | -.01 | .39** | .16* | .39** | .28** | .64** | -.14 | .76** | |  |  |
| 10. Age | 33.37 | 10.96 | | .08 | .14 | -.18* | -.05 | -.09 | .21** | -.06 | -.01 | -.01 |  |  |
| 11. Education Level | 3.61 | 1.07 | | -.19* | -.03 | .21** | .16* | .19** | .12 | -.24** | .06 | .03 | .15* |  |
| 12. Online Courses Taken | 6.34 | 13.6 | | .03 | .05 | .03 | .08 | .11 | -.02 | .00 | .04 | .02 | -.04 | .03 |

*Note. * indicates p < .05, and ** indicates p < .01. The scale ranges are 1 to 9 for data- and goal- driven effort judgements; 0 to 1 for test performance, prospective estimates and retrospective estimates; 1 to 5 for positive affect and negative affect; 1 to 7 for liking and future consumption;* *1 to 6 for education level, the one participant who reported “other” was not included in the correlations between education level and the other variables.*

**Appendix B**

**Table S3**

*Means (M), standard deviations (SD), and bivariate correlations between variables when effort was presented before affect.*

| Variable | *M* | | *SD* | 1 | 2 | 3 | 4 | 5 | 6 | 7 | 8 |
| --- | --- | --- | --- | --- | --- | --- | --- | --- | --- | --- | --- |
| 1. Data-Driven Effort | | 6.22 | 1.87 |  |  |  |  |  |  |  |  |
| 2. Goal-Driven Effort | | 6.84 | 1.51 | .35** |  |  |  |  |  |  |  |
| 3. Test Performance | | 0.58 | 0.23 | -.31** | -.11 |  |  |  |  |  |  |
| 4. Prospective Estimate | | 0.63 | 0.21 | -.50** | .06 | .29* |  |  |  |  |  |
| 5. Retrospective Estimate | | 0.52 | 0.24 | -.33** | -.05 | .61** | .65** |  |  |  |  |
| 6. Positive Affect | | 2.63 | 0.78 | .35** | .38** | -.25* | .02 | -.05 |  |  |  |
| 7. Negative Affect | | 1.45 | 0.44 | .34** | -.17 | -.16 | -.42** | -.10 | -.00 |  |  |
| 8. Liking | | 4.71 | 1.32 | -.07 | .34** | .03 | .29* | .05 | .50** | -.38** |  |
| 9. Future Consumption | | 4.53 | 1.70 | .08 | .44** | .05 | .26* | .09 | .61** | -.23 | .67** |

*Note. * indicates p < .05, and ** indicates p < .01. The scale ranges are 1 to 9 for data- and goal- driven effort judgements; 0 to 1 for test performance, prospective estimates and retrospective estimates; 1 to 5 for positive affect and negative affect; and 1 to 7 for liking and future consumption.*

**Table S4**

*Partial correlations between each type of effort report and test performance, metacognitive, and affect variables, controlling for the other type of effort report when effort was presented before affect.*

| Variable | Data-Driven Effort | Goal-Driven Effort |
| --- | --- | --- |
| Test Performance | -.29* | -.00 |
| Prospective Estimate | -.55** | .29* |
| Retrospective Estimate | -.33** | .08 |
| Positive Affect | .25* | .30* |
| Negative Affect | .44** | -.33** |
| Liking | -.21 | .39** |
| Future Consumption | -.09 | .44** |

*Note. * indicates p < .05, and ** indicates p < .01.*

**Table S5**

*Means (M), standard deviations (SD), and bivariate correlations between variables when affect was presented before effort.*

| Variable | *M* | *SD* | 1 | 2 | 3 | 4 | 5 | 6 | 7 | 8 |
| --- | --- | --- | --- | --- | --- | --- | --- | --- | --- | --- |
| 1. Data-Driven Effort | 6.24 | 1.87 |  |  |  |  |  |  |  |  |
| 2. Goal-Driven Effort | 6.69 | 1.71 | .45** |  |  |  |  |  |  |  |
| 3. Test Performance | 0.60 | 0.22 | -.47** | -.10 |  |  |  |  |  |  |
| 4. Prospective Estimate | 0.63 | 0.18 | -.34* | .19 | .45** |  |  |  |  |  |
| 5. Retrospective Estimate | 0.53 | 0.25 | -.37** | -.01 | .53** | .64** |  |  |  |  |
| 6. Positive Affect | 2.53 | 0.90 | .10 | .56** | .10 | .35** | .22 |  |  |  |
| 7. Negative Affect | 1.49 | 0.44 | .20 | -.01 | -.35** | -.44** | -.18 | -.12 |  |  |
| 8. Liking | 4.71 | 1.46 | .02 | .46** | .07 | .47** | .36** | .77** | -.17 |  |
| 9. Future Consumption | 4.45 | 1.73 | -.05 | .42** | .09 | .42** | .32* | .76** | -.20 | .85** |

*Note. * indicates p < .05, and ** indicates p < .01. The scale ranges are 1 to 9 for data- and goal- driven effort judgements; 0 to 1 for test performance, prospective estimates and retrospective estimates; 1 to 5 for positive affect and negative affect; and 1 to 7 for liking and future consumption.*

**Table S6**

*Partial correlations between each type of effort report and test performance, metacognitive, and affect variables, controlling for the other type of effort report when affect was presented before effort.*

| Variable | Data-Driven Effort | Goal-Driven Effort |
| --- | --- | --- |
| Test Performance | -.48** | .14 |
| Prospective Estimate | -.48** | .41** |
| Retrospective Estimate | -.41** | .19 |
| Positive Affect | -.21 | .59** |
| Negative Affect | .23 | -.12 |
| Liking | -.24 | .51** |
| Future Consumption | -.29* | .49** |

*Note. * indicates p < .05. and ** indicates p < .01.*
